# The value of serum creatinine as biomarker of disease progression in spinal and bulbar muscular atrophy (SBMA)

**DOI:** 10.1038/s41598-023-44419-6

**Published:** 2023-10-12

**Authors:** Lorenzo Blasi, Daniele Sabbatini, Andrea Fortuna, Giorgia Querin, Ilaria Martinelli, Sara Vianello, Cinzia Bertolin, Davide Pareyson, Maria Pennuto, Elena Pegoraro, Luca Bello, Gianni Sorarù

**Affiliations:** 1 Department of Neurosciences, Neuromuscular Center, University of Padova, 35128 Padua, Italy; 2 Institut de Myologie, I-Motion Adult ClinicalTrials Platform, Hôpital Pitié-Salpêtrière, Paris, France; 3 Neurology Unit, Department of Neurosciences, Azienda Ospedaliera Universitaria di Modena, Modena, Italy; 4 Clinical Genetics Unit, Department of Women and Children’s Health, University of Padova, IRP Città Della Speranza, Padua, Italy; 5 Department of Clinical Neurosciences, Unit of Rare Neurodegenerative and Neurometabolic Diseases, Fondazione IRCCS Istituto Neurologico Carlo Besta, Milan, Italy; 6 Department of Biomedical Sciences, University of Padova, Padua, Italy

**Keywords:** Biomarkers, Neurology, Pathogenesis

## Abstract

Serum creatinine has been indicated as a potential marker of motor function in SBMA and results form previous longitudinal studies pointed to its decline over time. This is a longitudinal retrospective study investigating creatinine changes over a 36-month-period in 73 patients with SBMA. Severity and progression of the disease was assessed according to serum creatine kinase (CK) values, manual muscle testing (MMT), SBMA functional rating scale (SBMAFRS) score**,** 6-min-walk test (6MWT) value, and spirometry (forced vital capacity, fVC%) obtained at the baseline and at each of the annual follow-up visits. Baseline serum creatinine concentrations positively correlated with 6MWT, the MMT megascore score of both the upper (ULM) and lower (LLM) limbs and SBMAFRS. No correlation was found with CK or fVC% values. Similar correlation results were achieved at all the subsequent time points. Longitudinal assessments conducted by the generalized estimating equations (GEE) method returned significant changes for SBMAFRS (− 1.41 points per year, *p* < 0.001), ULM and LLM (− 0.69, *p* = 0.01; and − 1.07, *p* < 0.001, respectively), 6MWT (− 47 m, *p* < 0.001) but not for creatinine (− 0.82, *p* > 0.05). We also observed that creatinine levels at baseline did not correlate with changes in the other measures from baseline at each annual visit. Our data do not support a role for serum creatinine as sensitive biomarker of disease progression, and possibily prognosis, in SBMA.

## Introduction

Spinal and bulbar muscular atrophy (SBMA), also known as Kennedy’s disease, is a rare, X-linked, late onset neuromuscular disorder^1^. SBMA is caused by a CAG repeat expansion in the first exon of the androgen receptor (AR) gene encoding for a polyQ tract, with a number of CAG repeats higher than 38 considered to be pathogenic^2^. The disease mainly manifests in adult males and is characterized by slowly progressive lower motor neuron (MN) degeneration in brainstem and spinal cord^3^, although there is accumulating evidence suggesting that polyQ-expanded AR primarily affects skeletal muscle as well^4–7^. The prevalent clinical feature of the disease is wasting and weakness of proximal limbs muscles in the lower limbs along with mild bulbar dysfunction and frequent length-dependent sensory neuropathy^3^. Multi-system involvement, mainly related to androgen insensitivity, integrates the clinical picture of the disease^8,9^.

There is no established therapy for SBMA and clinical trials conducted so far yielded overall unsatisfactory results^10^. The absence of sensitive measures to detect clinical changes in a slowly developing disease, such as SBMA, is considered a main concern in clinical trial design. Several biomarkers have been proposed to monitor SBMA progression, including functional scales or functional assessments, and electrophysiology studies^11^. Based on these measures, early recognition of subtle changes in disease status remains poorly exhaustive, even though the 6-min walk test (6MWT) may capture a 10% decline over 1 year^12^. More recently, promising results were obtained by skeletal muscle MRI which can demonstrate sensitive changes of fat infiltration over time^13,14^. Also wet biomarkers, i.e. bio-fluid molecules, are under study^15,16^ in the perspective of improving our capability to predict disease progression as well as response to therapy. Among these, creatinine has raised particular interest. Creatinine is a product of creatine phosphate catabolism in muscle^17^ from where it is released into the blood and freely filtered by the renal glomerolous^18^. Serum creatinine directly correlates with lean body mass in both healthy and diseased individuals^19–21^ and thus it may represent an indirect marker of muscle integrity and possibly function^22^. Recent studies have also brought to light the role of serum creatinine decrease as a marker of disease progression in amyotrophic lateral sclerosis, another MN disease^23,24^.

In SBMA patients, creatinine serum levels have been repeatedly reported to be reduced with values related to clinical parameters of disease severity, including functional outcome measures such as the 6MWT, the SBMA Functional Rating Scale^25,26^ and the Adult Myopathy Assessment Tool^13,27–29^, and muscle fat content on MRI^13^. Longitudinal assessment of creatinine concentrations was evaluated in two studies^13,28^, in which 32 and 17 SBMA patients were monitored for 3 years and 18 months, respectively. The findings of both studies were consistent with decreased creatinine levels over time, regardless of patients baseline characteristics^28^. In addition, Hijikata et al.^30^ observed that serum creatinine decrease begins more than 10 years before the clinical onset of SBMA and further decreases with clinical progression.

To further assess the value of serum creatinine as a disease progression marker in SBMA, we retrospectively evaluated clinical data form a wide cohort of patients with SBMA, pointing to correlations among creatinine values and other outcome measures according to both a cross-sectional and a longitudinal analysis.

## Methods

### Patients

This is a longitudinal retrospective study assessing clinical data obtained from annual visits of genetically confirmed Caucasian SBMA patients, referring to the Motor Neuron Disease Clinic of the University of Padova, between January 2014 and December 2019. We included only patients who were evaluated at least twice. The Local Ethics Committee approved the study and all study participants provided their informed consent in writing.

### Data collection

Patients’characteristics, including age at onset (described as subjective weakness in any part of the body, bulbar and/or spinal district) and at baseline visit, length of illness since onset of weakness, and number of CAG repeats, were collected. The severity and progression of the disease were assessed through the following measures obtained at the baseline visit and at each of the annual follow-up visit: 1. biochemical markers (serum creatine kinase, CK, and creatinine levels); 2. manual muscle testing (MMT) according to MRC score of the following muscles: deltoid, biceps brachii, triceps brachii, extensor carpi, opponens pollicis for upper limbs; iliopsoas, quadriceps femoris, anterior tibialis, and extensor hallucis longus for lower limbs; all muscles were tested bilaterally and cumulative scores for upper and lower limbs, namely upper limb megascore (ULM), range 0–50, and lower limb megascore (LLM), range 0–40, were used for statistical analysis; 3. SBMA functional rating scale (SBMAFRS) score^25,26^; 4. 6MWT distance (meters); 5. respiratory muscle function according to the forced vital capacity (fVC, expressed as percentage of predicted value). For each patient, glomerular filtration rate (GFR) and blood urea nitrogen were also annotated to monitor renal function.

### Statistical analysis

To verify any deviation from the normal distribution of the variables considered, the Shapiro–Wilk test was applied. Biochemical parameters were compared among data at different time-points (baseline, 12 months, 24 months, 36 months) using Wilcoxon Signed Rank Tests for repeated measurements on a single sample. Spearman’s rho correlation coefficient were assessed to verify a possible correlation between creatinine serum levels and clinical parameters at different time-points.

For longitudinal assessments, Generalized Estimating Equations (GEE) were used to evaluate all measure progression over time (i.e. per year). Spearman regressions were also performed to evaluate the correlation between the baseline creatinine values and the delta for each outcome (calculated as "outcome evaluation at the specific time point—outcome evaluation at baseline") in order to evaluate the prognostic effect. Finally, at each time point, the delta of each parameter was also compared with the delta creatinine at the specific time point. Statistical analyses were performed in R (R Foundation, version 4.0.2), with statistical significance set at P < 0.05 for all tests. To graphically represent data tidyverse and beeswarm packages were used.

### Ethics approval

This study was performed in line with the principles of the Declaration of Helsinki. Approval was granted by CESC (Comitato Etico per la Sperimentazione Clinica della Provincia di Padova), AOP1696.

## Results

Seventy-three SBMA patients were included in the study. Their mean age at disease onset was 43.2 years (median, 42 years; interquartile range [IQR], 37–50 years), mean age at baseline examination was 58.3 years (median, 58 years; IQR, 52–66 years; range, 38–79 years), after a mean disease duration of 15.2 years (median, 14 years; IQR, 9–20 years). The CAG repeat number ranged from 40 to 52 (mean 46; IQR, 44–47).

At the baseline visit, 57 patients (78%) had mild muscle weakness in all four limbs, 12 (18.4%) had mild to moderate weakness and required walking support, and 4 (6.1%) were using a wheelchair, being therefore unable to complete the 6MWT. None of them complained of significant respiratory or swallowing deficits.

Mean values of functional and biochemical measures at baseline and subsequent annual monitoring visits (12, 24 and 36 months), along with the number of individuals assessed at each time point, are reported in Table 1.

**Table 1:** Descriptive statistic of the studied clinical and biochemical parameters.

Parameter	Mean; [IQR] baseline	Mean; [IQR] at 12 months	Mean; [IQR] at 24 months	Mean; [IQR] at 36 months
Creatinine serum levels (umol/L)	53.82; [45.35–60.85] (71)	53.3; [27–61.4] (61)	51.55; [44.9–57.2] (65)	52.07; [44.45–60.25] (27)
6MWT (meters)	363.9; [275–450] (60)	317.1; [200–423] (60)	264.73; [95.25–417.75] (56)	226.5; [0–396.0] (35)
SBMAFRS	46.23; [43–51] (73)	45.23; [42–48.75] (70)	43.41; [39–48] (73)	42.15; [29–46] (48)
Megascore lower limbs	36.84; [19–39.5] (73)	35.99; [19–39] (70)	34.57; [33–38.5] (73)	33.77; [31.81–38] (44)
Megascore upper limbs	45.71; [43–49.5] (73)	44.6; [31–47.5] (70)	43.91; [41–48] (73)	43.80; [40.75–48] (41)
CPK (U/L)	1068; [481.2–1526.5] (62)	1065; [519–1445] (57)	1057; [554–1432] (55)	726; [518.5–853] (23)
% fvc	96.58; [88.50–106.50] (64)	95.43; [85.50–104.00] (51)	93.95; [82.50–102] (59)	82.90; [74.00–96.00] (14)

The number of patients at each time point is indicated in brackets.

*IQR* interquartile range, *6MWT* 6-min-walk test, *SBMAFRS* SBMA functional rating scale, *CPK* creatine kinase, *Fvc* forced vital capacity.

**Figure 1:**
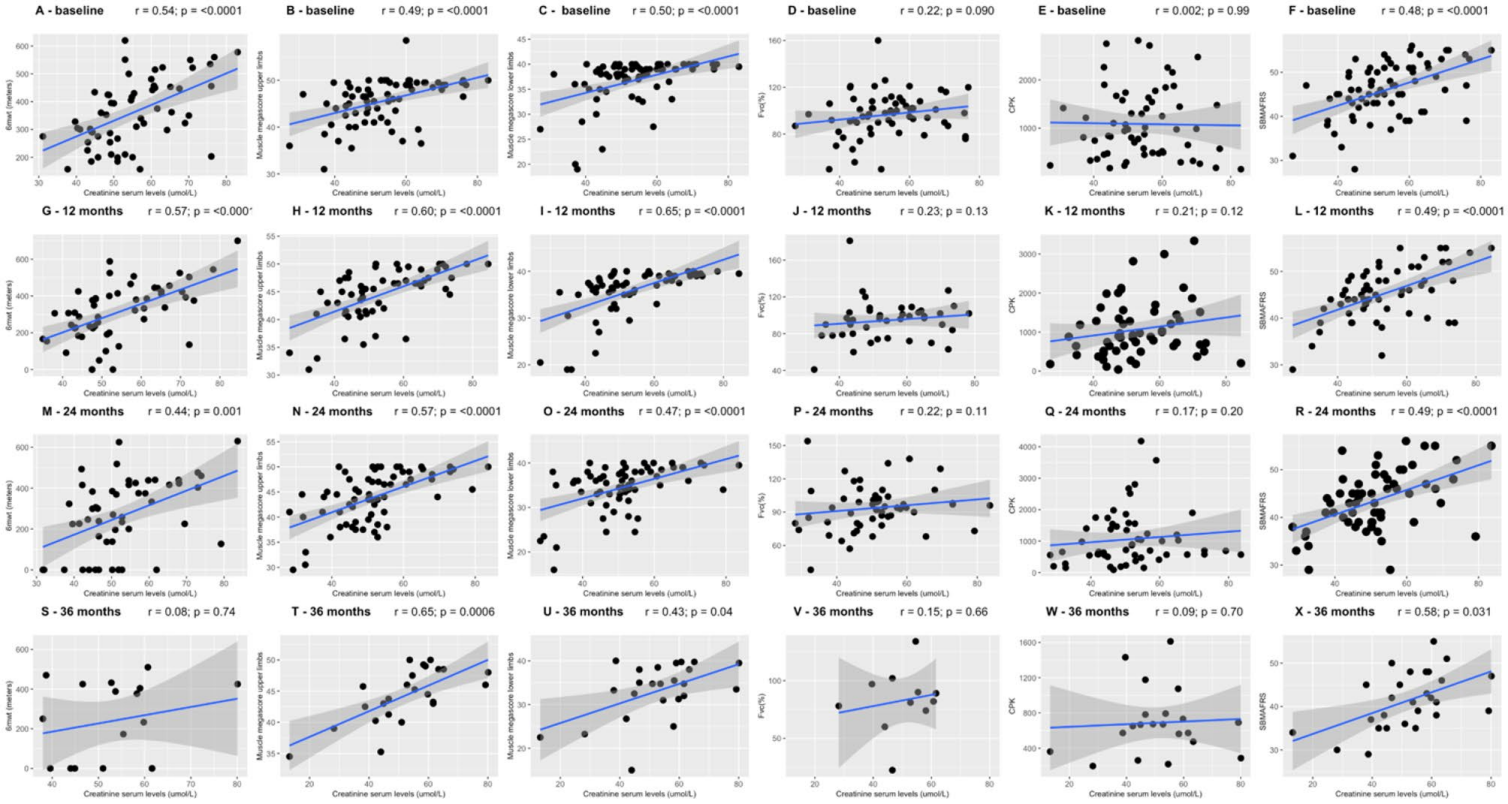
Scatter-plot between creatinine serum levels and clinical parameters at baseline. Spearman’s Rho coefficients (*r*) and p-values (*p*) from cross-sectional correlation are shown. *6MWT* 6-min-walk test, *Fvc* forced vital capacity, *CPK* creatine kinase.

Baseline serum creatinine concentrations correlated with the walked distance at the 6MWT *r* = 0.54; *p* = 1.244 × 10^–05^), the score of both ULM and LLM (*r* = 0.50, *p* = 1.77 × 10^–05^ and *r* = 0.49, *p* = 8.46 × 10^–06^, respectively) and SBMAFRS (*r* = 0.48, *p* = 1.854 × 10^–05^). No correlation was found with CK levels or fVC% values (Fig. 1). Similar results were achieved at all subsequent time points, although at the 36-month visit, a significant association between creatinine and 6MWT values was lost.

We did not observe any relation of creatinine with patients’age, disease duration, or CAG repeat number, whereas a significant correlation between disease duration and SBMAFRS (*r* = − 0.45; *p* = 0.00013), 6MWT (*r* = − 0.43; *p* = 0.0003), ULM and LLM (*r* = − 28; *p* = 0.023 and *r* = -0.50; *p* = 2.017 × 10^–05^, respectively) was noted. For each patient, renal function parameters were found within the normal range throughout the study period.

### Longitudinal analysis

Creatinine as well as the other functional measures were shown to decline over time (Fig. S1). However, GEEs calculations returned significant changes for SBMAFRS (− 1.41 points per year, 95% Confidence Interval [− 2.07, − 0.75], p = 3.80 × 10^–05^), ULM and LLM (− 0.69, 95% CI [− 1.21, − 0.17], p = 0.01; and − 1.07, 95% CI [− 1.64, − 0.50], p = 0.01, respectively), 6MWT (− 47 m, 95% CI [− 67.85, − 26.15], p = 1.01 × 10^–5^) but not for creatinine (− 0.82, 95% CI [− 2.41, 0.77], p = 0.30) and CK levels (− 72.6, CI [− 161.98, 16.78], p = 0.11). Creatinine levels at baseline did not correlate with changes from baseline in the other measures at each time point (except for ΔLLM at 12 months, p = 0.008) (Fig. S2). When looking at correlations between changes of creatinine levels and changes of functional measures (Fig. S3), throughout different time points, it seems of note that, at the latest time point of 36 months, a larger reduction in serum creatinine was associated with a larger drop in SBMAFRS scores (r = 0.57, p = 0.0020, Fig. S3, panel R); however, on the other hand, patients with larger creatinine reduction had more stable MRC megascores at the lower limbs (r = − 0.48, p = 0.018). Finally, we failed to observe any relation between the change at 36 months from baseline in all the outcome measures and patients’characteristics (patients’age, disease duration and CAG repeat number).

## Discussion

SBMA is a slowly worsening neuromuscular disease^28^ and a biomarker is not yet available to significantly track the disease progression in a period of time suitable for short-term trials^29^. Serum creatinine has been indicated as a potential marker of motor function in SBMA and results form previous longitudinal studies pointed to its decline over time in patients^13,28^.

In our retrospective study of 73 patients with SBMA, we confirmed a good and sustained correlation of creatinine with SBMAFRS, 6MWT and MMT although not with CK and FVC%. On the other hand, we also observed that creatinine values did not decrease significantly during the 36 months of observation, unlike SBMAFRS, 6MWT and MMT. Such a discrepancy between cross-sectional and longitudinal results of creatinine performance compared to the other outcome measures may possibily reflect the relative instability of creatinine concentrations due to mechanisms other than muscle mass/function. In fact, in addition to renal function, creatinine serum level is influenced by many variables including dietary intake or physical activity^19,31–34^. Similarly, Dahlqvist et al.^13^ observed that creatinine was stable among patients with protein levels below the reference range or increased in others during the 18-month observation period. Overall, these observations suggest a poor reliability of creatinine as a marker of short-term progression in SBMA.

In line with previous studies^28,29^, we confirmed that creatinine levels are unrelated to those of CK. Indeed, CK values are a marker of muscle injury rather than muscle function and they have also been reported not to correlate with functional parameters in SBMA^28,29^. Furthermore, CK values are vulnerable to a SBMA-specific impaired muscle metabolism of creatine^35^ and patient’s physical exercise prior to blood sampling^28^. Of interest, we reported a significant increase in CK levels in SBMA patients receiving beta2-agonist treatment who nevertheless showed improvement in motor performance^36^.

A relationship between creatinine and fVC values was also lacking, possibly because respiratory involvement may occur at advanced stages of the disease^37^ and, in addition, no patients of our cohort complained of respiratory issues.

Further, we assessed whether creatinine measurement could have prognostic significance. However, creatinine levels at baseline failed to predict changes in other measures over the observation period, nor was there a clear correlation observed between changes in creatinine and other measures compared to baseline.

This study has limitations including the retrospective design and the drop of patient number at the 36-month visit. As regards the latter point, the missing data basically belong to those patients who were initially followed at our center and who then moved to a nearest center following the recognition of other reference clinics across the country in accordance with the Italian SBMA Registry^38^. Therefore, we are confident that we can rule out any bias related to the disease course as the reason for the decline in patient ratings at 36 months.

In conclusion, our data do not support a role for serum creatinine as sensitive biomarker of disease progression in SBMA. Further studies that will also consider more recent outcome measures such as muscle MRI are warranted.

### Supplementary Information

Supplementary Figures.

## Data Availability

The datasets used and/or analysed during the current study available from the corresponding author on reasonable request.
