# Supplementary material for: The value of serum creatinine as biomarker of disease progression in spinal and bulbar muscular atrophy (SBMA)
Source: Sci Rep. 2023 Oct 12;13:17311. doi: 10.1038/s41598-023-44419-6 (PMC10570332; doi:10.1038/s41598-023-44419-6)
Supplement: Supplementary file 1 — Supplementary Figures. [file 41598_2023_44419_MOESM1_ESM.docx]

**Figure S1.** Longitudinal comparison of clinical parameters at baseline, 12, 24 and 36. The central location, scatter and dispersion of the observations are shown. A, Creatinine serum levels (umol/L); B, 6MWT (meters); C, SBMAFRS; D, Muscle megascore lower limbs; E, Muscle megascore upper limbs; F, CPK (U/L). 6MWT, 6-minute-walk test; SBMAFRS, SBMA functional rating scale; CPK, creatine kinase.


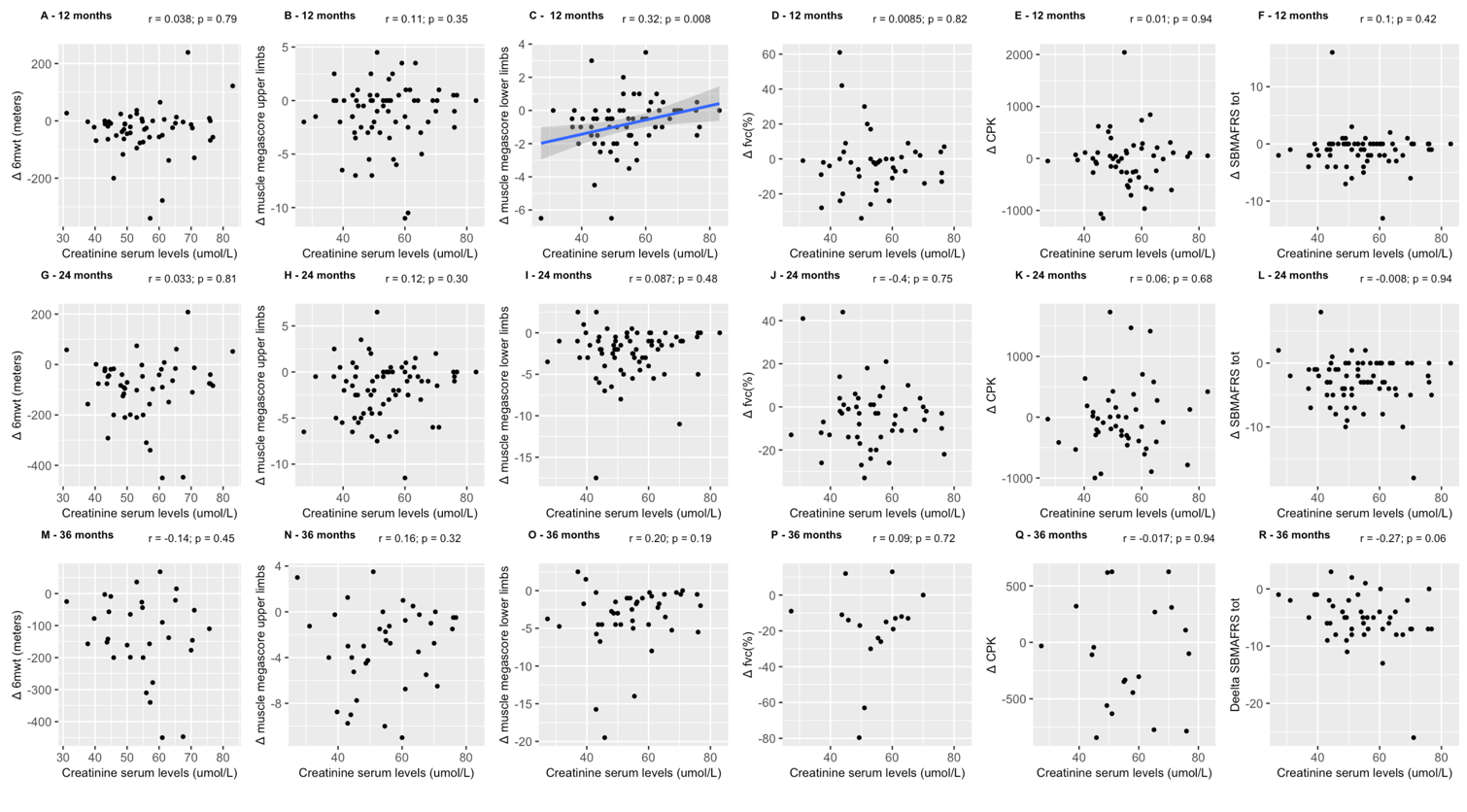


**Figure S2.** Scatter-plot between creatinine serum levels at baseline and Delta clinical parameters (calculated as "outcome evaluation at the specific time point - outcome evaluation at baseline"). Spearman’s Rho coefficients (r) and p-values (p) are shown. 6MWT, 6-minute-walk test; SBMAFRS, SBMA functional rating scale; Fvc, forced vital capacity; CPK, creatine kinase.


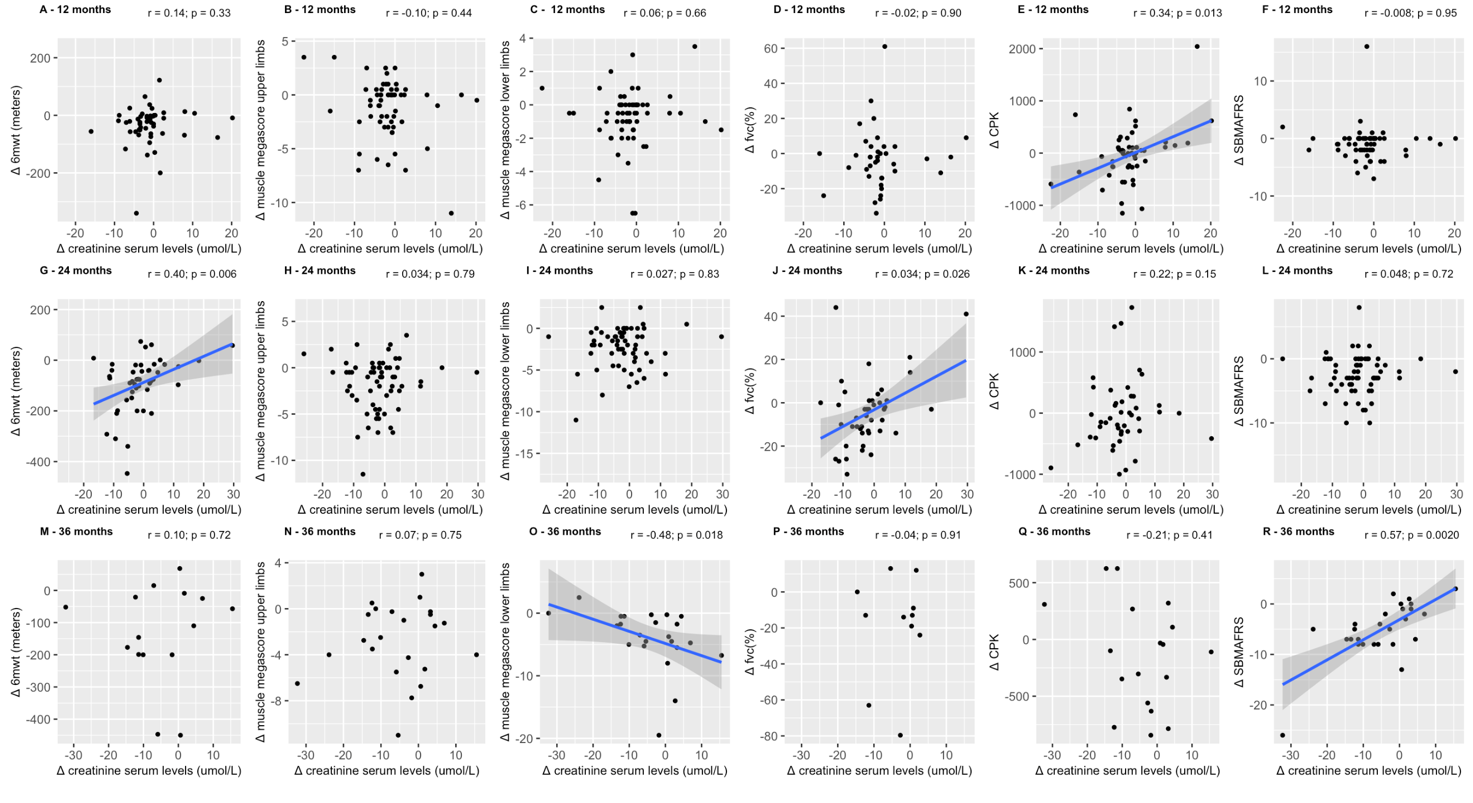


**Figure S3.** Scatter-plot between Delta creatinine serum levels and Delta clinical parameters (calculated as "outcome evaluation at the specific time point - outcome evaluation at baseline"). Spearman’s Rho coefficients (*r*) and p-values (*p*) are shown. 6MWT, 6-minute-walk test; SBMAFRS, SBMA functional rating scale; Fvc, forced vital capacity; CPK, creatine kinase.
